# Designing a minimum data set for the information management system (registry) of spinal canal stenosis: An applied‐descriptive study

**DOI:** 10.1002/hsr2.1671

**Published:** 2023-10-31

**Authors:** Javad Zarei, Ali Mohammadi, Mohamad Reza Akrami, Azar Jeihooni Kalhori

**Affiliations:** ^1^ Department of Health Information Technology, School of Allied Medical Sciences Ahvaz Jundishapur University of Medical Sciences Ahvaz Iran; ^2^ Department of Health Information Technology, School of Allied ‎Medical Sciences Kermanshah University ‎of ‎Medical Science Kermanshah Iran; ^3^ Department of Neurosurgery, School of Medicine Kermanshah University of Medical Sciences Kermanshah Iran

**Keywords:** Delphi technique, information management, minimum data set, registry system, spinal canal stenosis, spinal disease

## Abstract

**Background and Aims:**

Spinal canal stenosis is one of the most common vertebral column diseases, which can lead to disability. Developing a registry system can help in research on the prevention and effective treatment of it. This study designs a minimum data set (MDS) as the first step in creating a registry system for spinal canal stenosis.

**Method:**

The present research is of applied‐descriptive type, performed in 2022. First, the applicable data elements about the disease were selected from a vast range of English and Farsi references, including peer reviewed articles, academic books, credible websites, and medical records of hospitalized patients. Through the extracted data, the primary MDS plan was designed as a questionnaire. The validity of the questionnaire was conducted via asking the opinion of experts (neurosurgeons, physiotherapists, epidemiologists, and health information management specialists). Also, its reliability was calculated via Cronbach *⍺* coefficient, which was 86%. Finally, the MDS of the spinal canal stenosis national registry system (for Iran) was confirmed through a two stage Delphi technique. Data analysis was applied through descriptive statistics via SPSS21 software.

**Results:**

The proposed MDS is offered in two general sets of data: administrative and clinical. For the administrative data set, 40 data elements had been proposed, as five classes. Twenty‐six of them were confirmed. In the clinical section, 95 data elements had been proposed in 14 classes; 94 of which were finally confirmed.

**Conclusion:**

Since there is no spinal canal stenosis MDS available, this study can be a turning point in the standardization of the data on this disease. Moreover, these precise, coherent, and standard data elements can be contributed to improving disease management and enhancing the public healthcare quality. Also, the MDS proposed in this study can help researchers and experts, design a spinal canal stenosis registry system in other countries.

## 1 INTRODUCTION

Spinal canal stenosis refers to abnormal narrowing of the spinal canal, which may occur in the cervical, thoracic, and lumbar regions. The main reason for stenosis is degeneration of the spinal elements including intervertebral disc and ligament flavum.^1^, ^2^ Based on the criteria defined by the North American Vertebral Column Society, the most salient symptom of spinal canal stenosis is gluteal or lower limb pain, which aggravates with walking, standing, or back stretching, while improving with forward bending, sitting, or sleeping.^3^ The pain results from the constrained space available to neurovascular elements of the lumbar vertebral column.^4^

The degeneration becomes more intense with aging. The disease is one of the most important reasons of spinal injuries and diminished mobility among the people above 50 years old. This may be related to Osteoarthritis, which initiates changes in the vertebral column at age of 50.^5^, ^6^ Moreover, the prevalence of this disease increases at advanced ages. It is estimated that 400,000 people in the United States, mostly within the age range 60−69 years, have been affected by spinal canal stenosis. Forty‐seven percent of this age group has mild to moderate, and 19.7% of them have severe stenosis.^7^ Women are more at risk of developing spinal canal stenosis compared to men.^8^ In the United States, the healthcare costs associated with the back pain resulting from spinal canal stenosis have been estimated at 65 billion dollars annually. Furthermore, the indirect costs including loss of working days and production have been estimated at more than 170 billion dollars in total.^9^

Due to ageing of the world population, the incidence of degenerative spinal canal stenosis and its associated economic burden are growing.^2^, ^10^ This intensifies the necessity of research to improve healthcare and management of the disease. In 2021, the International Workshop on Diagnosis and Management of spinal canal stenosis was established with the support of the International Association of Studying Lumbar Vertebral Column to address some of the current challenges in spinal canal stenosis care provision.^2^ In addition, some registry systems have been created in recent years in different countries for registering vertebral column disorders, such as spinal canal stenosis. The examples include (NOR spine)^11^, ^12^ and the Swedish spine register (Swespine).^13^

One of the most essential stages in designing a registry system related to a special disease is determining it's minimum data set (MDS).^14^, ^15^ Launching a spinal canal stenosis population registry system provides the opportunity of recording data on a large scale during the routine care of patients. These data can be a valuable source for evidence‐based medicine and healthcare policies.^12^ In other words, MDS has a positive effect on the precise and comprehensive presentation of information on medical history, facilitation of healthcare plans, and healthcare provision quality enhancement as well as the duration of hospitalization and quality of life.^16^ Precise and coherent data in MDS are used for evaluating the incidence, prevalence, and burden of the disease at both national and international levels.^17^ MDS development is an important prerequisite to the collection of standards, integrated, and identical data about a disease. MDS provides high‐quality information for care providers by creating a national database.^17^ MDS can be used for collection and standardization of the data. It also eases the sharing and comparing data across various healthcare centers, as well as designing electronic health records of patients.^18^ In addition, MDS is a valuable source of information in improving patient care^14^ and for registry and reporting systems.^19^, ^20^

The proper selection of data elements is a major reason for the success or failure of registry systems.^20^ Through precise definitions of data elements, MDS should create a common language among all entities who are involved in registry and recording. In addition, it should guarantee the collection, analysis, reporting, and selection of essential data.^21^, ^22^ Further, MDS contributes to improving medical history files, data comparability, designing a data corpus, electronic exchange of data across various healthcare systems, and finally improving data quality.^21^, ^23^ According to the essence and application, the data elements of a disease can be categorized into administrative and clinical data. The administrative data usually include demographic and socioeconomic data, address, telephone number, and patient referral data, besides the main profession of the healthcare providers.^24^, ^25^ On the other hand, the clinical data differs depending on the type of disease. Generally, they include diagnosis, past medical history, laboratory results, medical imaging findings, healthcare interventions, disease progression and outcomes.^25^

In recent years, extensive studies have been done on the MDS in healthcare systems. Most of these studies, especially in Iran, have dealt with presenting a model for MDS development for a disease, injury, or group of patients.^19^, ^26^, ^27^, ^28^, ^29^, ^30^, ^31^, ^32^ However, two the best knowledge of authors, there is no specialized or comprehensive registry system for identifying patients with spinal canal stenosis, determining the disease outcome, and examining the effectiveness of its treatment methods. Thus, launching a registry system for spinal canal stenosis provides the possibility of determining the prevalence of the disease, the underlying factors contributing to the incidence of the disease, examining the complications resulting from the disease, exploring the effect of time of initiating the treatment, as well as various therapeutic interventions and the outcomes resulting from the treatment. This study expresses our experiences in the process of developing an MDS which can be useful for subsequent registrations especially in developing countries, which have limited infrastructures on information technology.

This study aims to propose a national MDS for the spinal canal stenosis registry system. However, it can be applied as a model for other regions, especially for developing countries. This data set can cover all influential factors in the health of these patients such as socioeconomic status, personal and familial history, disease‐developing conditions, comorbidities, diagnostic and therapeutic interventions, and healthcare outcomes.

## 2 METHODS

The applied study, was conducted descriptively and qualitatively in 2022 in Imam Reza hospital in Kermanshah, the West of Iran in four stages.

### 2.1 Study design

In the first stage, by studying the records of hospitalized patients diagnosed with spinal canal stenosis, their administrative and clinical data elements were extracted. In this regard, the list of cases was prepared from the hospital information system (HIS) based on the diagnosis code of spinal canal stenosis from ICD‐10. The records were extracted from the hospital file system based on the file number. Records were extracted by the study researcher and its data elements. The sources of the study were the files of hospitalized patients diagnosed with spinal canal stenosis (ICD‐10 code = M48.0). There were 200 cases with this diagnosis in the 2020−2021 time frame, all of which were studied.

The second stage included identifying data elements for risk factors, aggravating factors and long‐term consequences of the disease. Resources for this stage include papers, MDS in other countries, websites and clinical guidelines associated with the vertebral column diseases were investigated. Based on search strategy (Table 1), the relevant resources were extracted from internet based on the inclusion criteria. A comprehensive review of recovered resources was carried out until saturation.

**Table 1:** Search strategy for retrieving data elements of spinal canal stenosis.

Sites, criteria, keywords	Descriptions, characteristics
Websites	World health organization
Search engines	Google Scholar
Databases	PubMed; Scopus; SID; Mag Iran (through July 30, 2012)
Inclusion criteria	Literature in the English and Farsi language; scientific papers; annual reports; guidelines.
Exclusion criteria	Non‐peer‐reviewed papers, reports, and forms retrieved from personal weblogs; abstracts without accessible full text.
Keywords	“Spinal canal stenosis data element”; “Data element of Spinal canal stenosis”; “Spinal canal stenosis information management System”; “Minimum Data Set”; “Data Dictionary”; “Registry System or MDS”; “Spinal canal stenosis and Registry System.”

Abbreviation: MDS, minimum data set.

### 2.2 Data collection

In the stages one and two, data were collected using data extraction form that designed based on previous studies and advice of experts from health information management and neurosurgery. The collected data were first coded, then similar cases were grouped and the duplicate ones were eliminated. Finally, the data elements were categorized as the main group, data classes, data subclass, as well as values and data elements.

In the third stage, the proposed data elements are matched to the national health information systems in the Iran. The data elements were investigated considering the official health information systems such as the Iranian Health Electronic Record System (SEPAS) and Health Integrated System (SIB). The data collection instrument was a checklist, which was designed based on the findings of the first and second stages of the study. The checklist aimed to match the name and values of proposed data elements against the data elements offered in the mentioned systems, as well as to confirm the coding standard used for the data in them. The data collection was done via scrutinizing the mentioned systems and review of the available documents about their content. Based on the findings of previous stages of the study, the initial draft of the MDS of the spinal canal stenosis registry system was proposed. Next, this draft was provided to five experts including two neurosurgeons, two Health Information Management, Health Information Management, and one epidemiologist. After receiving the advices, the intended corrections were made. These corrections included changing the title of some data elements, changing the grouping of data elements, adding three new data elements, and removing two of them. Finally, based on the opinion of experts, a questionnaire was designed to determine MDS in both administrative and clinical departments. The questionnaire included administrative data elements (five classes) and clinical data elements (14 classes).

### 2.3 Analysis

The Delphi technique was employed for validating the developed MDS model of spinal canal stenosis national registry system. To measure the response of items, a five‐point Likert scale was used (very much, much, somehow, little, very little). Moreover, one open‐ended question (blank row) was set at the end of the data elements related to each class, to allow the experts to add any extra data deemed essential for the registry. For measuring the questionnaire reliability, Cronbach *⍺* coefficient was used. The coefficient was calculated for the main group and entire questionnaire. The coefficient obtained for the entire questionnaire was calculated at 86%.

To acquire the opinion of experts, the questionnaires were provided to them through in‐person visits or email. The experts included faculty members with specialties in health information management, neurosurgery, physiotherapy, and medical informatics. The expert selection criteria were knowledge related to spinal canal stenosis treatment and experience related to the registry of diseases and electronic health records. The selection of experts for the Delphi technique was done through the purposive sampling method. Overall, 25 experts were chosen in this step (Table 2) The criterion for selecting a data element in the questionnaire was 75% consensus of experts over that. In the first stage of Delphi decision‐making, the data elements with a consensus of less than 50% were removed. Also, the data elements within 50%−70% were reexamined in the second stage, and in case of acquiring more than 75% consensus, that element was considered the final element. Data analysis was done through descriptive statistics (frequency and mean) in SPSS21 software.

**Table 2:** Demographic characteristics of participants in the study.

Demographic characteristics	Frequency	Present (%)
Sex		
Male	15	60
Female	10	40
Education		
PhD	16	64
Physician	7	28
MSc	2	8
Specialty		
Neurosurgeon	7	28
Physiotherapist	3	12
Health information management	13	52
Medical informatics	2	8

This project was approved by the Ethics Committee of Ahvaz Jundishapur University of Medical Sciences under the code of ethics IR.AJUMS.REC.1400.311.

## 3 RESULTS

To prepare the desired MDS, two Delphi decision‐making stages were done. In the first stage, 135 data elements had been proposed, which included 40 elements for the administrative data and 95 for the clinical branch. In the groups of administrative data, 10 data elements were eliminated out of the 40 proposed ones, due to less than 50% consensus among the experts. The removed data elements were the phone number of patient's place of residence, patient's email, name of the service provider/service registrant, the surname of the service provider/service registrar, the healthcare center affiliation, the relevant medical sciences university, complete address of the healthcare center, the healthcare center email and its postal code. Moreover, 23 data elements related to the administrative data underwent a second opinion in the second stage of the Delphi study due to 50%−75% agreement among the experts. Among the 95 proposed elements for the group of clinical data, only one data element was eliminated, which was the test code. Further, the experts were asked again about 25 data elements, due to 50%−75% agreement over them. The data were related to the medical, individual, and family history of the patient, besides laboratory test, physiotherapy data, exercise recommendation and patient training, data on the disease course and the disease outcome. In the second stage of the Delphi method, a total number of 48 data elements were provided to the experts. Three of the administrative data elements were eliminated and 45 of the elements were approved. The final model included 120 data elements (27 administrative and 94 clinical data elements) which are summarized in Tables 3 and 4. The detailed list of classes and subclasses of administrative and clinical data elements in the final model are reported Tables 5 and 6.

**Table 3:** Summary of administrative data elements for minimum data set for spinal canal stenosis, underwent the Delphi method.

Data sections	Number of suggested data elements	The first round of Delphi method			The second round of Delphi method			The final number of data elements
		<50%, 50%−75%, >75%			<50%, 50%−75%, >75%			
Demographic data	8	0	4	4	0	0	4	8
Socioeconomic data	7	0	7	0	1	0	6	6
Residence address	5	2	2	1	0	0	2	3
Patient referral data	9	3	5	1	0	0	5	6
Healthcare identification data	11	5	5	1	2	0	3	4
Total	40	10	23	7	3	0	20	27

**Table 4:** Summary of clinical data elements for minimum data set for spinal canal stenosis, underwent the Delphi method.

Data sections	Number of suggested data elements	The first round of Delphi method			The second round of Delphi method			The final number of data elements
		<50%, 50%−75%, >75%			<50%, 50%−75%, >75%			
Signs and symptoms of the disease	15	0	0	15		None		15
Disability data due to illness	7	0	0	7		None		7
Diagnosis data	6	0	0	6		None		6
Individual and family medical history data	22	0	7	15	0	0	7	22
Medical imaging data	5	0	0	5	0	None		5
Laboratory tests	5	1	4	0	0	0	4	4
Electromyography (EMG/NCV)	4	0	0	4		None		4
Physiotherapy data	4	0	3	1	0	0	3	4
Exercise recommendations and patient education	5	0	5	0	0	0	5	5
Medication data	6	0	0	6		None		6
Surgery and invasive procedures	10	0	0	10		None		10
Discharge data	2	0	2	0	0	0	2	2
The course of the disease and the response to treatment	4	0	4	0	0	0	4	4
Total	95	1	25	69	0	0	25	94

**Table 5:** The data elements in each class and subclass of administrative category for MDS of spinal canal stenosis.

Main class	Subclass	Data elements
Demographic data		National identity number/passport number for foreign nationals‐medical record number‐patient name‐patient family‐father's names‐sex‐nationality‐age/date of birth
Socioeconomic data	Education	Educational degree (literacy, elementary, diploma, and …)
	Occupation	Employment status (part‐time/full‐time/unspecified)‐type of job (according to social security job list)‐job description‐average working hours per week‐difficult and loss‐making jobs (yes, no, unspecified)
Address		Type of residence (urban, suburban, rural), full residential address (country, province, city, town/village, region/neighborhood, …), mobile phone number
Healthcare identification data		ID, healthcare center name, healthcare center type
Patient referral data		Medical appointment‐type of visit‐date of data registration‐provider's/person's role (system manager, treating physician, consulting physician, referring physician, surgeon, physiotherapist, nurse, radiologist, data quality control officer, patient, patient's accompanying person)‐the specialist of the service provider/data recorder, The ID of the service provider/data recorder

Abbreviation: MDS, minimum data set.

**Table 6:** The data elements in each class and subclass of clinical category for MDS of spinal canal stenosis.

Main class	Subclass	Data elements
Signs and symptoms of the disease	Pain	Type of pain, location, pain severity/scale, radiation, conditions that aggravate pain, conditions that relieve pain
	Other signs and symptoms	Date of the first symptoms‐numbness in the limbs‐tingling in the limbs‐early fatigue‐weakness in the limbs‐disturbance in the way walking‐balance problem‐sexual intercourse disorder‐urine and feces excretion disorder
Disability data due to illness		Oswestry disability index score‐limitation in movement (mobility)‐limitation in self‐care‐limitation in interaction (getting along)‐limitation in life activity‐limitation in participation (participation)‐limitation in activities and participation code based on ICF
Diagnosis		General types‐location‐exact anatomical location of the involved vertebra or vertebrae (lumbar, cervical, thoracic)‐other spine disorders (degenerative, spinal osteoarthritis, osteoporosis)‐accompanying neurological disorders (cauda equine, myelopathy)‐diagnosis code/diagnoses based on ICD
Individual and family medical history		Height‐weight‐degree of curvature of the lumbar arch (over 35 degrees)‐underlying disease (yes, no, unknown)‐name of the underlying disease (type 2 diabetes, etc.)‐code of the underlying disease based on ICD‐duration of the disease‐is the disease under treatment or not?‐Relationship of the underlying disease with spinal canal stenosis‐history of trauma to the spine‐nature of the injury (type of injury and anatomical location)‐history of other spine diseases‐name of the disease‐history of related congenital muscular‐skeletal abnormalities‐family history of spine diseases‐proportion of the affected person‐history of previous treatment for spinal diseases‐type of treatment‐exercise‐type of exercise (name and weekly average)‐smoking and drug or alcohol use‐type of substance consumed
Medical imaging		Date‐type of medical imaging (X‐rays, MRI, CT scan, CT myelogram)‐imaging procedure name‐imaging procedure code‐diagnosis (impression)
Clinical laboratory tests		Date‐test group‐test name‐test result
Electromyography	EMG and NCV nerve	Date‐EMG and NCV operation name‐muscles and organs examined‐diagnosis (impression)
Physiotherapy		Date of the first session‐number of sessions‐physiotherapy service name‐physiotherapy service code
Exercise recommendation and patient education		Date of training/order‐the type of recommendation to educate the patient‐implementation schedule/implementation method‐evaluation of the result of the action‐other complementary and supportive interventions (such as psychological and occupational medicine measures)
Medication		Prescription date‐prescription drug name (generic/brand names, drug form, dosage)‐drug code‐drug dosage
Surgical/invasive procedures		Date of procedure–the type of surgical/invasive procedure‐procedure name‐procedure code‐exact anatomical position‐prosthesis use‐prosthesis name‐surgery complications occurred‐name/description of postoperative complication‐code of postoperative complication (according to ICD)
Complication surgical procedure	Early complication	Surgical site infection‐opening of stitches‐numbness in the legs‐ occurrence of a blood clot in the lower limb (embolism)
	Late complication	Prosthetic fracture‐replacement of screws and rods‐incontinence in defecating and urinating‐fibrous tissue formation at the surgical site‐drop foot‐neurogenic lameness (pain and numbness in the back when sitting or when standing or bending forward)
Discharge status		Discharge date (for hospitalized patients)‐length of stay (hospitalized patients)
The course of the disease and the response to treatment		Evaluation date‐the result of evaluating the course of the disease and the process of response to treatment‐description of the evaluation/follow‐up‐the final result of treatment performed

Abbreviation: MDS, minimum data set.

## 4 DISCUSSION

In the present study, a MDS for spinal canal stenosis has been presented for Iran. It is divided into the administrative and clinical data groups. Most studies on the MDS have also used this classification. The examples include MDSs presented for C‐section anesthesia information management system,^16^ traumatic brain injuries,^33^ speech therapy,^34^ cardiac electrophysiology,^35^ patients suffering from pelvic floor disorders,^36^ postmortem computed tomography report for anthropological biological profiling,^37^ and COVID‐19 registry.^18^ The reason for this classification has been the different nature and application of administrative and clinical data. The first general group of data included administrative data. These data are usually used for identifying patients, registering the healthcare institute or provider specifications, service costs, and administrative reports.^18^

Given the applications mentioned in the present study, the administrative data sets for the spinal canal stenosis registry system included five classes capturing demographic data, socioeconomic data, address, healthcare identification data, and patient referral data. In the class of demographic data, eight data elements had been proposed, all being approved by the experts. The only difference between this class with other similar studies in Iran ^16^, ^17^, ^18^, ^19^, ^38^ on proposed data elements was the passport number of foreign citizens, due to referral of patients from Iraq and Afghanistan for treating spinal canal stenosis in Iran. Considering the importance of registering socioeconomic data in patients with spinal canal stenosis, for the socioeconomic data class, seven data elements were proposed, all being approved by the experts except for ethnicity. Some of the data proposed in this class had differences from similar studies.^36^, ^38^ Considering the effect of type of occupation on the severity of disease as well as the patient's response to the treatment, four data elements were considered for describing the patient's job. Given the wide extent of occupations, for precise determination of the type of job, it was proposed that the job type be chosen based on the official list of Social Welfare Organization Jobs in Iran.

Given the study aim, which was a population registry system, one of the practical data for studying the incidence and prevalence of the disease across different regions was the patient's address. Thus, five data elements had been proposed for the address class, with three of them being approved by the experts. Two of its data including the type of residence and complete address of the patient per each country, province, and so forth had been considered for supporting the epidemiological studies of the disease across different regions. The data and classification of their values were similar to the MDS of COVID‐19 registry^18^ and the MDS of the gestational diabetes registry.^14^ The two next classes of the administrative data set belonged to the healthcare identification data and patient referral data. Overall, nine out of 20 proposed data elements were approved by the experts. These data had been proposed for registering the information of healthcare providers and patient referrals, which were similar to other studies.^14^, ^18^, ^26^

The second group of data belonged to clinical aspects. A total of 94 data elements had been proposed in 14 classes, with only one remaining unapproved by the experts. The aim of the classification and proposal of data in each class was to provide important information about the disease risk factors, diagnosis, treatment, and evaluation of the treatment response process, and disease outcome. The first class of clinical data covered the disease signs and symptoms. Since pain is weighed as the most important symptom of the disease,^39^, ^40^ a separate subclass and six data elements were considered for the pain evaluation. Apart from the pain‐associated data elements, eight data elements were also considered for other common signs and symptoms of the disease. In chronic diseases such as spinal canal stenosis, the severity and number of symptoms and signs of the disease play a key role in selecting the treatment plan and disease outcome determination (such as postoperative assessment).^40^ Thus, in the registry of vertebral column diseases, a section is dedicated to pain assessment.

In addition to the severity of signs and symptoms of spinal canal stenosis, the extent of disability resulting from the disease is also very important in the treatment plan and evaluation of its effectiveness. In particular, in lumbar spinal stenosis, disability evaluation becomes further important.^40^ Thus, a special class had been considered for the data elements of the disease disability. The first data element belonged to the Oswestry disability index score. It is used as a routine standard in the evaluation of pain and disability in vertebral column diseases.^41^ In addition to the Oswestry disability index score, based on the WHO Disability Assessment Schedule (WHODAS), five data elements were proposed for evaluating the limitation in activities and participation. WHODAS is a well‐known instrument for evaluating health and disability worldwide.^42^ Some studies have also shown the effectiveness and validity of this instrument for evaluating the disability resulting from vertebral column diseases.^43^ In our study, the reason behind proposing the evaluation of disability through WHODAS was the feasibility of patient disability status coding with ICF or V chapter (Supporting Information section for functioning assessment) ICD‐11. Ahmadi et al. also in designing the MDS of disability health information management system, had proposed ICF for disability coding.^24^ The next class belonged to diagnosis, and included six data elements. Considering the WHO schedule for the implementation of ICD‐11, as well as its pilot implementation in Iran,^44^ attempts were made to set the data elements for registering sufficient details associated with diagnosis, in line with the codes related to spinal canal stenosis and other accompanying disorders.

The individual and family medical history included 22 data elements. It had been proposed first for supporting the registry of individual and family risk factor for spinal canal stenosis. The second aim was to register other underlying diseases, which can negatively affect the course or treatment of the disease. In comparison to similar registry systems such as NOR spine^12^ and Spine Tango,^11^ more data elements have been considered for registering the disease risk factors. For example, in Spine Tango, height, weight, and cigarette smoking had been considered as risk factors.^11^

In the clinical data, three classes captured para‐clinical diagnostic data elements (medical imaging, clinical laboratory tests, and electromyography). The medical imaging data such as MRI have great value in diagnosing spinal canal stenosis. In our study, five data elements had been proposed for medical imaging, which was similar to other studies conducted in Iran. In our study, the aim of presenting this format for diagnostic data elements such as medical imaging was to facilitate the data collection and registry in a health electronic information system (according to the action code data element). However, in the radiology form of the Spine Tango registry, numerous data elements had been considered for registering complete details of the imaging findings.^45^

For registering the treatment process data in patients with spinal canal stenosis, four data classes of physiotherapy, exercise recommendation and patient education, surgical/invasive procedures, and medication had been proposed. The data proposed in these four classes were similar to the MDS of disability^24^ and the MDS of the HIS.^29^

Treatment of spinal canal stenosis depends on the clinical conditions of the patient, undergoing physiotherapy, patient education, exercise, epidural steroid injections, and surgical treatment.^39^ One of the data elements in these classes was the intended therapeutic intervention code. Use of the code allows for a standard registry of healthcare as well as data exchange between the disease registry information system software and other health information systems such as Electronic Health Record.^46^ Considering the plan of Iran's Health Ministry for EHR development,^47^ a data registry using a standard coding system facilitates data exchange between the information management system of spinal canal stenosis and HER in the future. In the class on surgery and invasive interventions, it was proposed to use ICHI for the coding of interventions. Some studies have shown that ICHI, in comparison to other intervention classification systems, has a better performance in registering the patient's information in the electronic health record.^48^

For a complete registry of the surgical intervention data, in addition to the surgical/invasive procedures class, a separate class had also been considered for registering the complication surgical procedure. In the international registry of vertebral column Spine Tango,^11^ the postoperative complications data elements had been propounded. As the spinal stenosis is most common among the elderly, surgical operation is associated with more risk of unwanted complications.^49^ Complication surgical procedure was classified in two subclasses of early and late complications. The presence of these data elements can contribute to complete registry of surgical interventions.

The discharge status class has been proposed for registering the information on the treatment outcome in hospitalized patients, including two data elements of discharge data and duration of hospital stay. The duration of hospital stay is a kind of useful data for comparing various surgical techniques and evaluating their outcomes.^50^ The last data class in the clinical data group belonged to the course of the disease and the response to treatment. The aim of this class was to register the treatment follow‐up and response to treatment information.

## 5 CONCLUSION

The MDS proposed in this study is designed to support a comprehensive registry of administrative and clinical data of patients with spinal canal stenosis. In this MDS, attempts were made to first emphasize the epidemiological aspects of the disease, and provide the possibility of registering and collecting patient data for various research through a clinical approach. Moreover, the possibility of data exchange between the registry information system software and HER is considered, besides using new classification systems such as ICHI. In this regard, two stage of Delphi technique was conducted by the cooperation of 25 experts in various related fields including neurosurgeons and physiotherapist besides the specialists in health information management and medical informatics. The developed model included 120 data elements in two major branches of administrative data and clinical data, each involved 27 and 94 data elements, respectively. The application of this MDS can contribute to establishing a spinal canal stenosis registry system in Iran. It can also provide a higher quality registration of patient information in different healthcare centers.

## AUTHOR CONTRIBUTIONS

**Javad Zarei**: Conceptualization; funding acquisition; project administration; supervision; validation; writing—review and editing. **Ali Mohammadi**: Data curation; methodology; project administration; writing—review and editing. **Mohamad Reza Akrami**: Conceptualization. **Azar Jeihooni Kalhori**: Data curation; formal analysis; investigation; software; writing—original draft.

## CONFLICT OF INTEREST STATEMENT

The authors declare no conflict of interest.

## ETHICS STATEMENT

The ethics committee of Ahvaz Jundishapur University of Medical Sciences, Ahvaz, Iran approved the study with the ethical approval code: IR.AJUMS.REC.1400.311. All patients were guaranteed their information would be kept confidential.

## TRANSPARENCY STATEMENT

The lead author Azar Jeihooni Kalhori affirms that this manuscript is an honest, accurate, and transparent account of the study being reported; that no important aspects of the study have been omitted; and that any discrepancies from the study as planned (and, if relevant, registered) have been explained.

## Data Availability

The data that support the findings of this study are available from the corresponding author upon reasonable request. [Corresponding author or manuscript guarantor] had full access to all of the data in this study and takes complete responsibility for the integrity of the data and the accuracy of the data analysis.
